# Simultaneous induction of dispersed and clustered DNA lesions compromises DNA damage response in human peripheral blood lymphocytes

**DOI:** 10.1371/journal.pone.0204068

**Published:** 2018-10-31

**Authors:** Lei Cheng, Beata Brzozowska, Alice Sollazzo, Lovisa Lundholm, Halina Lisowska, Siamak Haghdoost, Andrzej Wojcik

**Affiliations:** 1 Centre for Radiation Protection Research, Department of Molecular Biosciences, the Wenner-Gren Institute, Stockholm University, Stockholm, Sweden; 2 Biomedical Physics Division, Faculty of Physics, University of Warsaw, Warszawa, Poland; 3 Institute of Biology, Jan Kochanowski University, Kielce, Poland; Northwestern University Feinberg School of Medicine, UNITED STATES

## Abstract

Due to its ability to induce DNA damage in a space and time controlled manner, ionising radiation is a unique tool for studying the mechanisms of DNA repair. The biological effectiveness of ionising radiation is related to the ionisation density which is defined by the linear energy transfer (LET). Alpha particles are characterised by high LET, while X-rays by low LET values. An interesting question is how cells react when exposed to a mixed beam of high and low LET radiation. In an earlier study carried out with human peripheral blood lymphocytes (PBL) we could demonstrate that alpha radiation X-rays interact in producing more chromosomal aberrations than expected based on additivity. The aim of the present investigation was to look at the mechanism of the interaction, especially with respect to the question if it is due to an augmented level of initial damage or impaired DNA repair. PBL were exposed to various doses of alpha particles, X-rays and mixed beams. DNA damage and the kinetics of damage repair was quantified by the alkaline comet assay. The levels of phosphorylated, key DNA damage response (DDR) proteins ATM, p53 and DNA-PK were measured by Western blotting and mRNA levels of 6 damage-responsive genes were measured by qPCR. Alpha particles and X-rays interact in inducing DNA damage above the level predicted by assuming additivity and that the repair of damage occurs with a delay. The activation levels of DDR proteins and mRNA levels of the studied genes were highest in cells exposed to mixed beams. The results substantiate the idea that exposure to mixed beams presents a challenge for the cellular DDR system.

## Introduction

While contemplating on the stability of hereditary properties, Erwin Schrödinger argued that the stability of the genetic material must be “of the almost absolute” and that mutations are due to rare quantum jumps in the gene molecule [1]. Today, it is well known that the DNA is labile and suffers constant damage both from endogenous and exogenous factors. Endogenous DNA damage originates mainly from errors in DNA replication and oxidative stress, while exogenous damage originates from environmental, occupational and medical exposure to chemical and physical genotoxins [2]. Despite these attacks, the genome remains stable thanks to efficient DNA repair mechanisms. However, the capacity and fidelity of DNA repair is critical in retarding the processes of aging and preventing a wide number of pathologies, including cancer [3]. Indeed, disorders associated with deficient DNA repair are associated with a high incidence of cancer and accelerated aging [4].

A wide range of DNA repair mechanisms has evolved to cope with the various forms of DNA damage. Among the lesions, DNA double strand breaks (DSB) play a prominent role because they disrupt the DNA molecule and their repair is often error prone, leading to chromosomal rearrangements and possibly genomic instability [5]. DSBs can occur at different levels of complexity, the degree of which is inversely correlated with the likelihood of their correct repair [6]. A complex DSB is defined as being composed of at least three single-strand breaks within 10 base pairs and other DNA damage types nearby such as oxidised bases and DNA-protein crosslinks [7].

Ionising radiation is a particularly potent inducer of DSB [8]. It evokes its detrimental effect on cells by localized deposition of energy that is sufficiently large to eject orbital electrons from atoms. For a given amount of energy that is deposited inside a cell, its spatial distribution determines the biological effectiveness of the radiation. Gamma radiation or X-rays deposit the energy in a scattered manner randomly inside a cell, while alpha particles and heavy ions deposit the energy in a dense, clustered manner along the particle track [9]. The ionisation density is described as linear energy transfer (LET, given in keV per μm) and while gamma radiation and X-rays are characterized by low LET, alpha particles and heavy ions are characterized by high LET values, especially at the end of their tracks when the particles are stopped by matter [10]. An important consequence of the difference in ionisation density is that low LET radiations mainly induce simple DSB while high LET radiations induce many complex DSB [9].

Ionising radiation is abundant on Earth so that it is a constant source of damage to the DNA but the level of natural background radiation is strongly variable [11]. In certain situations people are exposed to a mixed field of high and low LET radiation, for example in areas with high levels of both the alpha emitting radon-222 and the alpha plus gamma emitting radium-226 [12] or during air travel, where gamma radiation occurs concomitantly with neutrons and protons [13]. Mix beam exposure also takes place during certain forms of cancer radiotherapy. During intensity modulated radiotherapy (IMRT) and proton therapy, patients to unwanted neutrons [14–16]. The neutron dose might be high enough [17, 18] to cause relevant cancer effects. This is a concern because increasing rates of cure lead to the appearance of such long term side effects like secondary cancers [19]. Neutrons interact with matter leading to the production of charged particles which are characterised by high LET. Thus, patients are exposed to a mixed beam of low (primary beam) and high (neutrons) LET radiation.

Despite the frequent mixed beam exposure scenario, its impact to DNA has not been studied extensively. Of special interest is the question how the cellular DNA repair machinery copes with a simultaneous induction of dispersed simple and clustered, complex DSBs. Do the complex DSBs engage the DNA damage response machinery to such a degree that the dispersed, simple DSB are not sensed or repaired properly? Or do cells primarily focus on repairing the dispersed, simple DSBs so that the repair of complex DSBs is compromised?

We have constructed a dedicated mixed beam exposure facility, where cells can be simultaneously exposed to alpha particles and X-rays [20] and could demonstrate that the radiations interact in producing synergistic cellular effects, meaning higher than expected based on additivity of the two radiation types [21–25]. The mode of interaction is not clear, but using U2OS cells and live microscopy we recently showed that cells react to mixed beams by concentrating the DNA damage response (DDR) protein 53BP1 in selected foci, possibly preferentially at sites of clustered DNA damage that are formed early during the irradiation process. Moreover, mixed beam-induced foci showed a low degree of mobility, possibly contributing to augmented misrepair of damage, especially of complex DSBs.

The synergistic effect of mixed beams was not seen with all cell types and end points [20], but we consistently observed a higher than expected level of cytogenetic damage in human peripheral blood lymphocytes (PBL) [21, 22]. The aim of the present investigation was to look more closely at the mechanism of the synergistic effect in PBL, especially with respect to the question if it is due to an augmented level of initial damage or impaired DNA repair. The level of DNA damage and the kinetics of damage repair was quantified by the alkaline comet assay. In addition, the levels of phosphorylated, key DDR proteins were measured by Western blotting and the mRNA levels of 6 radiation-responsive genes were measured by qPCR.

## Materials and methods

### Blood donors, blood collection and irradiation

Experiments carried out in the framework of this study were approved by the regional ethical committee in Stockholm (permit number 2010/27-31/1). Participation in the study was voluntary and participants gave their informed, verbal consent. Fresh peripheral blood was collected shortly before irradiation by venepuncture from 3 healthy, non-smoking male donors aged 30, 34 and 55. A single experiment was always performed with blood from a single donor. For the comet assay experiments, whole blood was diluted 1:1 with RPMI 1640 medium (Sigma-Aldrich, R5886, Stockholm, Sweden), kept on ice for 10 min and exposed or sham exposed to alpha particles, X-rays or a mixed beam of both radiations on pre-chilled, round polyamide (PA) discs (155 mm in diameter, custom-constructed in the Institute for Energy-JRC, Petten, Netherlands) as described earlier [21]. 250 μl of diluted blood was placed at the centre of a PA disc, covered with a 2.5 μm thick Mylar foil lid and spread over the disk to form an even layer. For analysing the expression of selected proteins by Western blotting, isolated peripheral blood mononuclear cells (PBMC) were irradiated as described in a dedicated section below.

The exposures were performed with an irradiation facility consisting of an alpha irradiator (^241^Am source, 50.0 ± 7.5 MBq, Eckert & Ziegler Isotope Products GmbH, Germany) and an YXLON SMART 200 X-ray tube (operating at 190 kV, 4.0 mA, no filtering), which allows exposure irradiation of cells with alpha particles and X-rays separately and simultaneously [20]. A movable shelf in the alpha irradiator was used to position the cells on a PA disc at defined distance from the alpha source. The cells were exposed to alpha particles when the shelf was in the top position. The dose rate of alpha radiation was 0.223 Gy/min at the entrance to the cell suspension and the average LET was 90.9 ± 8.5 keV μm^-1^. The dose rate of X-ray was 0.052 Gy/min in the top position and 0.068 Gy/min in the bottom position of the movable shelf. For studying the dose response relationship, cells were exposed to doses of 0 to 2 Gy. DNA repair kinetics and the expression levels of selected proteins were analysed following a dose of 2 Gy. The mixed beam was composed of 50% alpha particles and 50% X-rays. Combined exposure always started with both the X-ray machine on and the exposure dish in the top position (alpha radiation “on”). After reaching the desired alpha dose, the dish was moved to the bottom position (alpha radiation “off”) and X-irradiation continued until the desired X-ray dose was reached.

After exposure, blood was transferred from the PA disc to an Eppendorf tube. For analysing the dose response relationship, the blood was kept on ice. For analysing DNA repair, the blood was further diluted 1:5 with RPMI 1640 medium supplemented with 20% foetal bovine serum (Gibco, Invitrogen, Stockholm, Sweden), 100 U/ml penicillin and 100 μg/ml streptomycin (Sigma-Aldrich, P4333, Stockholm, Sweden) and incubated at 37°C for 0, 15, 30, 60, 120 and 180 min. After incubation, blood was chilled on ice until further processing.

### Alkaline comet assay

Alkaline comet assay was performed with whole blood as described by [26] with slight modifications. The irradiated, diluted blood was warmed up quickly in a 37 °C water bath and mixed with 2% low melting point agarose (2-Hydroxyethylagarose, Type VII, Sigma-Aldrich, A4018) at a final concentration of 1%, dropped on a microscopy slide pre-coated with 0.5% high melting point agarose (Type I-A, Sigma-Aldrich, A0169), covered with a coverslip and placed on an ice-cold plate to solidify. Duplicate slides were made for each individual sample. After solidification, coverslips were removed and slides were immersed in a cold lysis buffer (2.5 M NaCl, 100 mM Na_2_EDTA, 10 mM Tris, pH 10 and 1% Triton X-100) for 1 h at 4 °C under slight shaking. Following a brief rinsing in cold distilled water, slides were randomly positioned in a horizontal electrophoresis tank filled with fresh, cold electrophoretic buffer (1 mM Na_2_EDTA and 300 mM NaOH, pH > 13) and kept for 40 min for DNA unwinding. After electrophoresis (1 V/cm, 30 min, 4 °C), slides were washed with 0.4 M Tris (pH 7.5) 3 times and stained with 4’,6-diamidino-2-phenylindole (DAPI). Each slide was coded and analysed blindly. Per slide, 50 randomly selected comets (100 comets per sample) were captured at 200 × magnification using a fluorescent Nikon Eclipse E600 (Tokyo, Japan) microscope. Images were analysed by the Comet Assay II (Kinetic Imaging, Liverpool, UK) software. Percentage of DNA in the comet tail was chosen as the measure of DNA damage and referred to as relative tail intensity (RTI).

### Western blot

Experiments were performed with isolated PBMCs. Whole blood was diluted 1:1 with Hank´s Balanced Salt Solution (HBSS, Sigma Aldrich, H9394, UK) overlaid on Ficoll-Paque Premium solution (GE Healthcare, 17-5442-02, Uppsala, Sweden) and centrifuged at 400 × g for 35 min. The layer containing PBMCs was removed and washed twice with HBSS by centrifugation with 100 × g for 10 min. After that PBMCs were suspended at a density of 8–10 x 10^6^ cells/ml in complete medium composed of RPMI 1640 medium (Sigma Aldrich, R5886, St Luis, MO, USA) supplemented with 20% FBS (HyClone, Thermo Fisher Scientific, Waltham, MA, USA) and 1% PenStrept (10.000 U penicillin and 10 mg streptomycin/ml, Sigma Aldrich).

250 μl of a PBMC suspension was irradiated with 2 Gy X-rays, alpha particles or mixed beams on a PA dish as described above. Control cells were kept on PA dish for 15 min. After exposure, PBMCs were suspended in complete medium and cultured in a 5% CO_2_, humidified, 37°C incubator for 1 h or 3 h. After incubation and subsequent centrifugation, PBMC pellets were lysed using the RIPA buffer (50 mM Tris-HCl (pH 7.4), 150 mM NaCl, 0.5% Igepal, 5 mM EDTA (pH 8.0), 0.1% SDS) supplemented with PhosStop and Protease inhibitor cocktail tablets (Roche Diagnostics GmbH, Mannheim, Germany). Protein concentration was estimated using DC^™^ Protein Assay kit (Bio-Rad Laboratories, Hercules, CA, USA).

Electrophoresis was carried out by loading 40 μg of protein per sample per well on NuPage 3–8% Tris-acetate gradient gels in NuPage Tris-acetate running buffer (Novex, Life technologies, CA, USA), at 150 V for 65 min. The proteins on the gel were then transferred to a nitrocellulose membrane (Thermo Scientific, Rockford, USA) and the membrane was blocked at room temperature for 1 h using a blocking buffer (Odyssey Blocking Buffer (LI-COR, Cambridge, UK) and Tris-buffered saline containing 0.05% Tween (TBST), 1:1). The following primary antibodies were used: pDNA-PKcs (pSer2056; SAB4504169, Sigma-Aldrich, 1:300), pATM (pSer1981; SAB4300100, Sigma-Aldrich, 1:500), phospho-p53 (pSer15; #9284, Cell Signaling Technology, 1:500) and GAPDH (G8795, Sigma-Aldrich, 1:20 000). The membrane was incubated overnight at 4°C with primary antibodies diluted in the blocking buffer. Next day it was washed with TBST and probed with secondary antibodies for 1 h at room temperature. The secondary antibodies were either infrared dye-conjugated goat anti-rabbit or donkey anti-mouse secondary antibodies (LI-COR, Cambridge, UK, 1:15000 diluted in TBST), according to the primary antibodies. The membrane was scanned and quantified using the Odyssey Fc Infrared Imaging System (Li-COR). Three independent experiments were performed.

### Gene expression analysis by qPCR

RNA was extracted using the E.Z.N.A. Total RNA Kit I (Omega Bio-tek) from isolated PBMCs after 2 Gy irradiation of X-rays, alpha particles and mixed beams following different time points incubation (4h, 24h and 48h). The method of PBMCs isolation was described above. cDNA was synthesised from 250 ng RNA using the High-Capacity cDNA Reverse Transcription Kit (Thermo Fisher Scientific) with random hexamer primers. Primers, cDNA and PowerUp^™^ SYBR^™^ Green Master Mix (Thermo Fisher Scientific) were mixed and real time PCR reactions were performed in duplicate on a LightCycler^®^ 480, starting at 50°C for 2 min and 95°C for 2 min, followed by 40 cycles of 95°C for 15 s, 60°C for 15 s and 72°C for 1 min. No template control reactions were used to identify PCR contamination. The 2^−ΔΔCt^ method was used for calculation of relative expression and melting curve analysis was used for testing primer specificity. Primers used were: BBC3 for: TACGAGCGGCGGAGACAAGA, BBC3 rev: GCAGGAGTCCCATGATGAGATTGTAC; FDXR for: TGGATGTGCCAGGCCTCTAC, FDXR rev: TGAGGAAGCTGTCAGTCATGGTT; GADD45a for: ACTGCGTGCTGGTGACGAAT, GADD45a rev: GTTGACTTAAGGCAGGATCCTTCCA; XPC for: GCTTGGAGAAGTACCCTACAAGATGGT, XPC rev: GGCTTTCCGAGCACGGTTAGA; MDM2 for: TATCAGGCAGGGGAGAGTGATACA, MDM2 rev: CCAACATCTGTTGCAATGTGATGGAA; CDKN1A for: CCTGGAGACTCTCAGGGTCGAAA, CDKN1A rev: GCGTTTGGAGTGGTAGAAATCTGTCA. For 18S, sequences are given in [27].

### Statistical analysis

The dose response relationships and repair kinetic curves were fitted with the help of CurveExpert Professional version 2.6 (https://www.curveexpert.net). The functions of best fits to the data are shown in Eqs 1 and 2.

Dose response:

$$y=a(1-e^{-bx})$$ (1)

Where *y* is the percent DNA in the comet tail, *x* is the dose and *a* and *b* are the fit coefficients.

Repair kinetics:

$$y={\left(a-bx\right)}^{-\frac{1}{c}}$$ (2)

Where *y* is the relative percent DNA in the comet tail, *x* is the time point and *a*, *b* and *c* are the fit coefficients. The values of the fit coefficients are given in the S1 Table.

The distributions of cells according to percent DNA in the tail were fitted by the Gnuplot 5.0 (www.gnuplot.info) with a Weibull distribution described as:

$$y=(\frac{a}{b^{a}})x^{a-1}exp(-{\left(\frac{x}{b}\right)}^{a})$$ (3)

where: *x* represents the percent DNA in the comet tail, *a* is the shape parameter and *b* is the scale parameter. The values of the fit coefficients are given in the S2 Table.

The significance of interaction between alpha particles and X-rays in inducing DNA damage was tested by constructing envelopes of additivity [28]. An envelope of additivity is constructed from iso-effect plots which are calculated based on assuming isoadditivity or heteroadditivity of the damage levels induced by the components of a combined exposure [29]. Whether the components of a combined exposure interact or not is judged by analysing the position of an observed effect with respect to an envelope: the position inside the envelope indicates additivity, below the envelope—synergism, and above the envelope—subadditivity. Envelopes are constructed for three levels of effect: low, median and high.

Differences between the levels of phosphorylated proteins were tested by the unpaired, 2-tailed Student’s t-test. Differences between levels of initial damage were tested by one-way ANOVA Tukey test.

## Results

**Fig 1:**
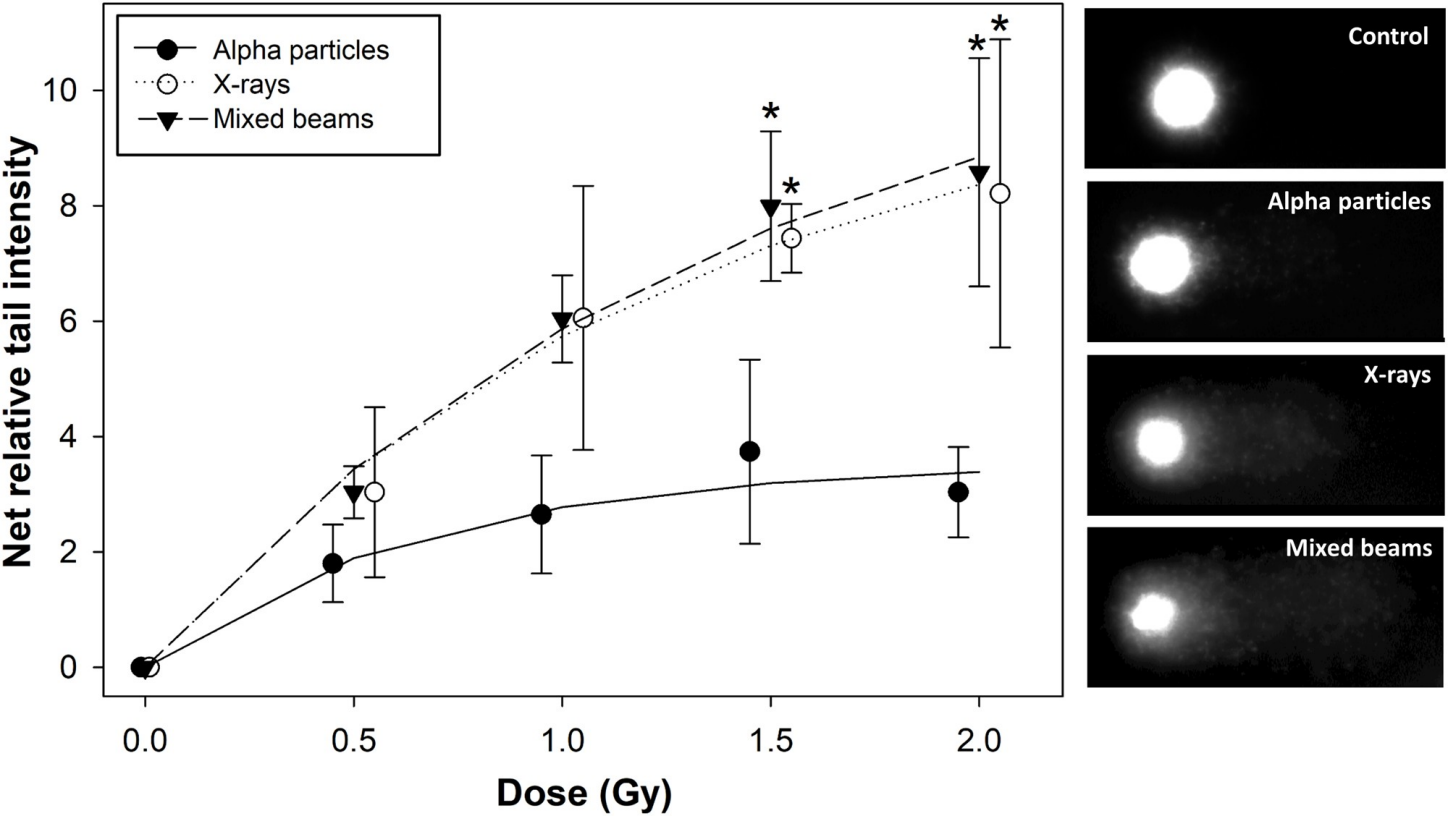
Dose response relationships for initial DNA damage. Net relative tail intensity: percent DNA in the tail minus control. Symbols represent mean results from 3 independent experiments. Error bars represent standard deviations. *: significant (p<0.05) difference to alpha particles (one way ANOVA). Symbols are nudged for transparency. Exemplary images of comets following exposure to 0 Gy (control) and 2 Gy of alpha particles, X-rays and mixed beam are shown to the side of the graph.

Comet assay was used to assay the damage induction and repair following mixed beam radiation. Percentage of DNA in the comet tail was chosen as the measure of DNA damage and referred to as relative tail intensity (RTI) as suggested by [30]. Mean control levels of initial damage from 3 repeats per radiation type were 3.67 ± 1.01 for alpha particle experiments, 2.11 ± 0.55 for X-rays and 2.58 ± 0.63 for mixed beams (± symbolises standard deviations). Control RTI were subtracted from respective radiation-induced RTI and the relationships between the net DNA damage, measured immediately after radiation exposure, are shown in Fig 1 together with exemplary images of observed comets.

X-rays and mixed beams induced very similar levels of damage, while alpha particles showed the weakest dose response. For all radiation types, the dose response relationship showed a tendency to saturate with increasing dose. The effect was strongest for alpha particles leading to a significantly lower level of damage as compared to X-rays and mixed beams at doses of 1.5 and 2 Gy. The fitting parameters to the data (S1 Table) allowed calculating the relative biological effectiveness (RBE) of alpha radiation and of mixed beams. The values for net tail intensity of 1 and 3 are, respectively, 0.18; 0.34 for alpha radiation and 1.0; 0.98 for mixed beams.

Envelopes of additivity were constructed in order to verify whether the initial level of damage after mixed beam exposure is different than expected by assuming an additive action of alpha particles and X-rays [28]. To this end two isobolograms were plotted, one assuming isoaddition and the other assuming heteroaddition [29]. The isobolograms form an envelope and the location of the measured event inside the envelope indicates additivity, below the envelope—synergism and above—subadditivity. In general, envelopes of additivity are constructed for a dose inducing a low, medium and high level of the measured event [29]. In the present investigation, envelopes were constructed for RTI of 1, 2 and 3.5.

**Fig 2:**
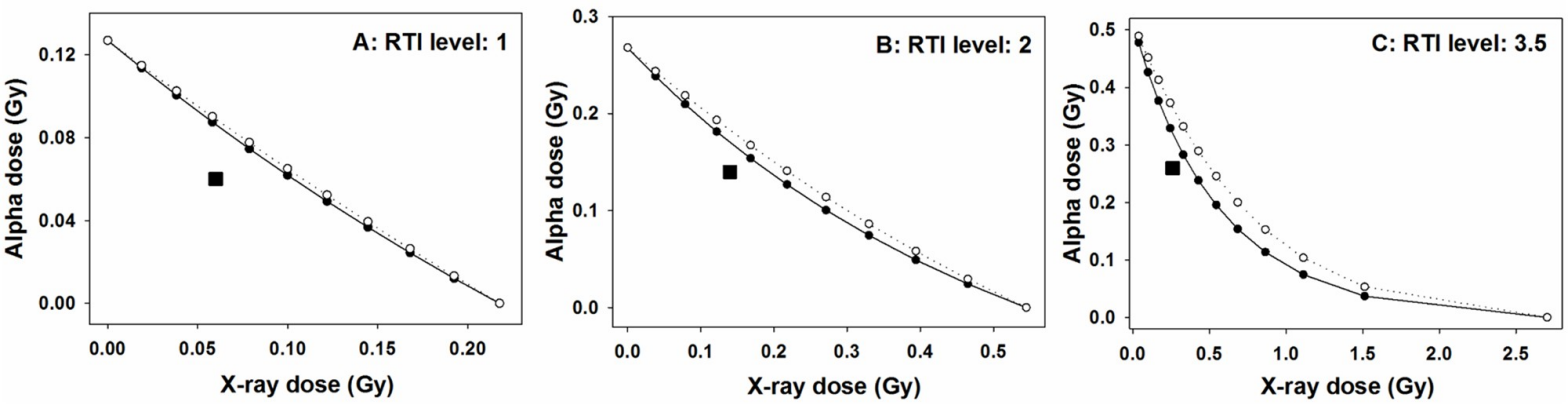
Envelopes of additivity (circles and lines) for different levels of relative tail intensity (RTI), calculated for initial damage. Squares represent the observed RTI in cells exposed to both alpha particles and X-rays at dose levels indicated, respectively, on the X and Y axes. The isobolograms and the observed RTI were derived from fits to dose-response relationships.

The results are shown in Fig 2. Irrespective of the level of damage, the mixed beam of alpha particles and X-rays always induced a higher than expected level of RTI, indicating an interaction (synergism) of alpha particles and X-rays in inducing DNA damage. The level of interaction was inversely related to the level of damage, with the strongest interaction at a low level of damage (panel A) and the weakest at the highest level of damage (panel C). This result is an outcome of the saturating dose response curves shown in Fig 1.

**Fig 3:**
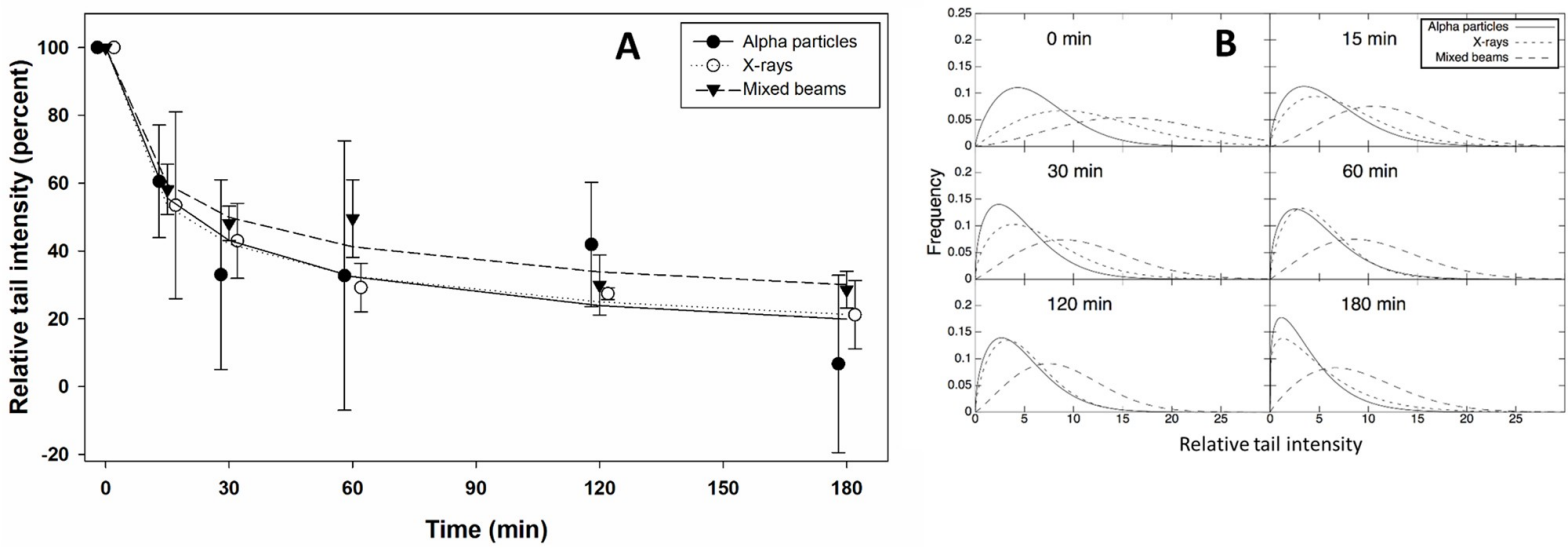
A: Repair kinetics following a dose of 2 Gy. Symbols represent mean results from 3 independent experiments. Relative tail intensity (percent): RTI normalized to initial level of damage. Error bars represent standard deviations. Symbols are nudged for transparency. B: Distributions of relative tail intensities at various time points post exposure.

DNA repair kinetics were analysed following a dose of 2 Gy. RTI was estimated 15, 30, 60, 120 and 180 min post exposure. In view of the fact that the level of RTI was significantly lower in cells exposed to alpha particles as compared to X-rays and mixed beams, the results were normalised to the initial level of damage, allowing a comparative analysis of the decline of DNA damage with time. The results are shown in Fig 3A. No significant differences in the repair curves were observed between the treatments, although cells exposed to mixed beams appeared to repair DNA damage less efficiently than cells exposed to X-rays and alpha particles. This result is supported by the RTI distributions which are shown in Fig 3B which demonstrates that the repair of DNA damage in cells exposed to mixed beams occurred with a delay. The RTI values in Fig 3B are gross values (controls not subtracted).

**Fig 4:**
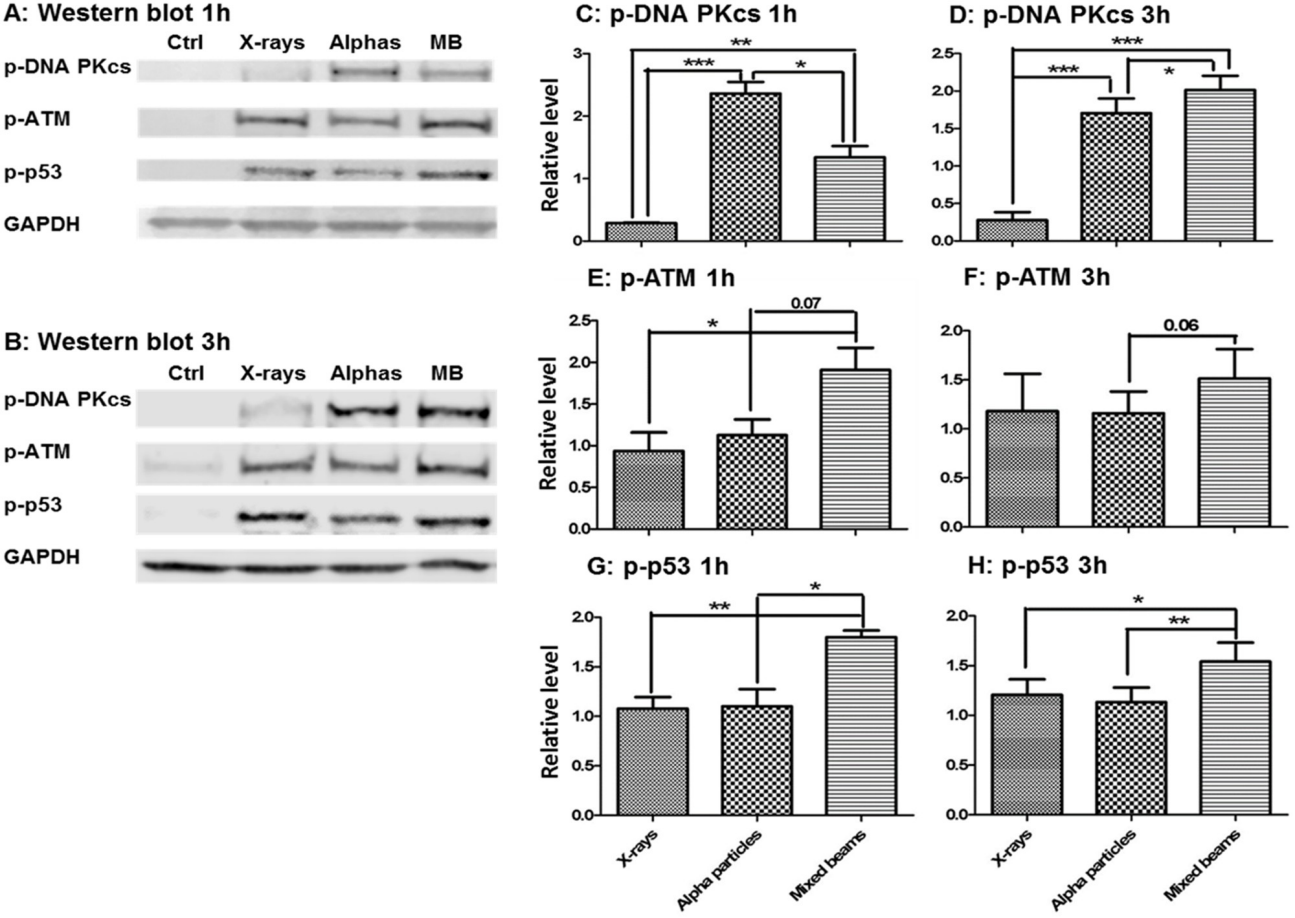
Levels of phosphorylated proteins DNA-PKcs, ATM and p53 1h and 3h post exposure. The relative level was calculated as fold increase in relation to GADPH and the average of each protein intensity of all samples. Mean results from 3 independent experiments for 1h and 5 independent experiments for 3h. Error bars represent standard errors. Asterisks represent significant differences at the level of * < 0.05, **<0.01 and ***<0.001 (Students t-test).

Western blot analysis was carried out to verify whether the results obtained with the comet assay were related to the activity of key proteins involved in DDR. The levels of phosphorylated proteins DNA-PKcs (pSer2056), ATM (pSer1981) and p53 (pSer15) were analysed 1h and 3h post exposure. The results are shown in Fig 4. Exposure to mixed beams resulted in the highest activation of ATM and p53 at both time points. A somewhat different result was obtained with DNA-PKcs which, 1h post exposure, was most strongly activated following exposure to alpha particles. However, 3h post exposure its level following exposure to mixed beams was significantly higher than after alpha particles.

**Fig 5:**
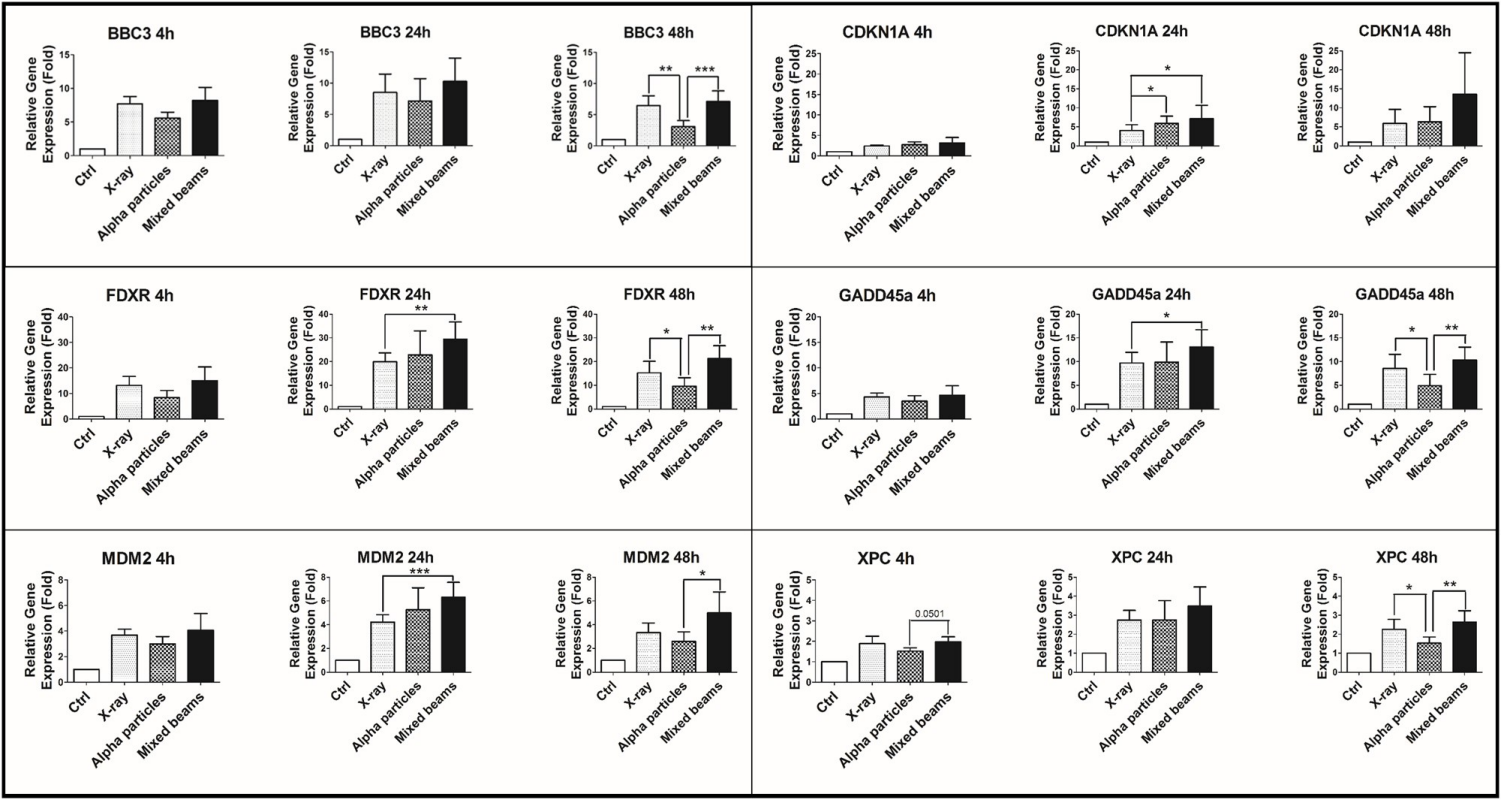
mRNA levels of DNA damage-responsive genes in human PBL after 4h, 24h and 48h incubation following 2 Gy exposure with X-rays, alpha particles and mixed beams. Error bars represent standard errors. Asterisks represent significant differences at the level of * < 0.05, **<0.01 and ***<0.001 (Students t-test).

In order to verify how the high activation by mixed beams of DNA-PKcs, ATM and p53 was reflected in the expression of DNA damage responsive genes, the mRNA levels of the following genes were measured 4, 24 and 48h post irradiation: GADD45A, CDKN1A, MDM2, XPC, BBC3 and FDXR. The results are shown in Fig 5. Generally, the levels of mRNA measured were lowest 4h post exposure, highest at 24h post exposure and intermediate at 48h post exposure. Although not significant in the case of every analysed gene, the mRNA level of all genes was highest in cells exposed to mixed beams, especially at 48h post exposure. No clear general pattern of difference was observed between cells exposed to X-rays and alpha particles.

## Discussion

The results of the investigation demonstrate that alpha particles and X-rays interact in inducing DNA damage above the level predicted by assuming additivity and that the repair of damage occurs with a delay. Moreover, the activation levels of the key DDR proteins ATM (a DNA damage sensor [31]), p53 (the master switch in damaged cells [32]) and DNA-PK (the key protein in non-homologous end joining [33]) were highest in cells exposed to mixed beams (albeit only at 3h post exposure for DNA-PK). Also the mRNA levels of 6 radiation-responsive genes, the transcription of which is regulated by p53 [34, 35], were highest in cells exposed to mixed beams up to 48 h post exposure, supporting the above results. The outcome of the study corroborates our earlier findings which show that alpha particles and X-rays interact in producing micronuclei [21] and chromosomal aberrations [22] in PBL above the level expected by assuming an additive action of both radiation types.

The significance of interaction between alpha particles and X-rays in inducing DNA damage was tested by constructing envelopes of additivity [28]. This approach must be used whenever the dose response curves of the single radiation components of a mixed beam are not linear. The reason for this is that nonlinearity of a dose response implies interaction of damage induced by a single agent [29]. Hence, a possible interaction of two agents is impossible to predict. The problem is solved by constructing two isobolograms under the assumption that both agents do interact (isoaddition) or they do not (heteroaddition). Both isobolograms are plotted together with an experimentally derived data point and the significance of interaction is assessed based on the position of the data point with respect to the isobolograms [28, 29]. However, the observation of a significant interaction does not provide any information regarding its mechanism.

As suggested earlier [24], the interaction of alpha particles and X-rays can occur via various mechanisms. Firstly, it is possible that the action of both radiation types will lead to an increase of LET and, consequently, of DNA damage complexity. Secondly, it is possible that exposure to high LET radiation will engage the DDR machinery to such a degree that the additional damage induced by the low LET radiation will not be repaired properly. The results of the current study do not give a decisive answer as to which mechanism is most likely responsible for the synergistic action of alpha particles and X-rays. However, in our opinion, arguments which are described below, favour the second mechanism.

We used the alkaline comet assay to study the induction and repair of DNA damage. The advantage of the comet assay is its high sensitivity and the possibility to analyse individual cells [30]. The assay measures relaxation of DNA supercoiling and is, in its standard alkaline version, not able to detect clustering of DNA damage. Alpha particles are known to induce a high level of clustered and complex damage [36, 37] and if the comet assay was able to detect them, then the dose response curve for alpha particles should be steeper than that for X-rays. Such relationships was observed for chromosomal aberrations [22]. In contrast, we observed a shallower dose response curve for alpha particles than X-rays resulting in RBE values lower than 1. This is in good agreement with results obtained by analysing gamma-H2AX foci [23, 38] and 53BP1 foci [25] and can be explained by the lower number of cells per unit dose hit by alpha particles as compared to X-rays: while a dose of 1 Gy alpha radiation corresponds on average to 5 alpha tracks per cell nucleus, 1 Gy of X-rays corresponds on average to 2000 photon tracks per cell nucleus [Brzozowska et al, unpublished). If mixed beams induced a level of DNA clustering beyond that of alpha particles, then the RTI values should be lower or, given the X-ray component which made 50% of the total dose, similar to RTI induced by alpha particles. This was not the case: the initial level of mixed beam-induced DNA damage was the same as following exposure to X-rays alone and significantly higher than that induced by alpha particles. Also the levels of phosphorylated DDR proteins were highest in cells exposed to mixed beams, while, with the exception of DNA-PKcs, they were similar in cells exposed to alpha particles and X-rays. A similar result was observed for the levels of mRNA of radiation-responsive genes. So taken together, the results do not suggest that exposure to mixed beams leads to an increase of LET and, consequently, of DNA damage complexity.

In order to verify if exposure to high LET radiation in a mixed beam scenario engages the DDR machinery to such a degree that the additional damage induced by the low LET radiation will not be repaired properly, we analysed the kinetics of DNA repair. In order to eliminate the confounding differences in the level of damage for each radiation type, the results were presented as percent of initial damage. The analysis revealed a lagging repair of damage induced by mixed beams. This is particularly visible at the level of RTI distributions, where a fraction of cells exposed to mixed beams persistently showed an augmented level of RTI which fits well with the elevated gene expression levels which was detectable until 48h post exposure. The results support the outcome of our recent investigation on the induction and decay of 53BP1 foci which form around DNA double strand breaks [25, 39]. Cells reacted to the combined exposure to alpha particles and X-rays by concentrating the 53BP1 protein in large foci, possibly forming at sites of clustered DNA damage. This happens at the cost of small foci forming at sites of dispersed, simple damage. Taken together, these findings provide evidence for a high engagement of the DNA damage response machinery by the complex damage induced by alpha particles so that the repair of additional damage induced by the low LET radiation is delayed.

The results are interesting not only from the perspective of how cells react to DNA damage of various complexities but also from the perspective of radiation protection. They suggest that, per unit dose, the biological effect of mixed radiation beam may be higher than expected based on a simple sum of single doses coming from radiations of various qualities, even if these are multiplied by appropriate radiation weighting factors [40]. The impact of the finding on transfer of cancer risk among cohorts exposed to different radiation types has been extensively discussed in [24]. Here a short discussion is given on why the results may also be important for the medical use of radiation in IMRT and proton therapy.

In proton therapy, an ongoing debate deals with the appropriate value of the relative biological effectiveness (RBE) inside and ahead of the planned treatment volume [41]. Experimentally, the RBE is determined using in vitro cell systems, where stray radiation [18] inside the patient body is not taken into consideration. If protons interact with the produced neutrons and gamma radiation, then the experimentally determined RBE is lower than that the RBE inside the patient body. In IMRT, X-rays of energies greater than approximately 6 MeV can generate neutrons by (γ,n) reactions through interactions with the components of the accelerator and the treatment room, as well as inside the patient body [42, 43]. The unwanted neutron dose to the patient might be high enough (in the range of several hundred mSv) to cause relevant carcinogenic effects [17]. The RBE of neutrons as a function of its energy is well characterised [40] and can be used to predict the risk of second cancers [43]. However, if neutrons interact with gamma radiation, then the risk of secondary cancers is underestimated. It should also be mentioned that, as recently suggested, deficient repair of complex DNA damage may be linked to constant DDR triggering and the continuous activation of the immune system which may also potentiate the risk of cancer [44].

From the perspective of radiotherapy, our data also point to a future possibility in combining alpha emitters or other types of high LET radiation with conventional photon therapy. An alpha emitter targeted to the skeleton for treatment of bone metastases of castration-resistant prostate cancer (Xofigo, ^223^RaCl_2_) was approved in 2013 [45]. Several clinical trials have been performed in different tumour types with alpha emitters such as ^213^Bi, ^221^At and ^225^Ac bound to monoclonal antibodies towards tumour-specific proteins (reviewed in [46–49]) The mixed beam synergistic effect and the partly separate toxicity profile and range of alpha emitters and X-rays might allow for a lowered dose of both to create a therapeutic window. A previous trial using a mixture of neutrons and photons suggested no significant advantage for mixed beam radiation therapy over photon radiation therapy alone for head and neck cancer patients, however there was a better response in a subgroup with lymph node metastasis [50]. Three fractions of photons and two fractions of neutrons were given each week, so most likely, they were not given concomitantly. A low LET combination of photons and electrons was promising using Monte Carlo dose calculations, sparing organs at risk considerably better than each therapy alone [51].

It should be remarked that the above considerations are valid provided that the mixed beam effect occurs in all cell types. Our experience does not allow drawing a decisive conclusion about whether this is the case. We observed synergism in PBL with cytogenetic damage as the endpoint [21, 22], human lymphoblastoid TK6 (clonogenic cell survival) [24] and human bone osteosarcoma epithelial (U2OS) cells [25, 39] (DNA repair foci). In contrast, we registered additivity in human VH10 cells [23] (DNA repair foci) and in AA8 Chinese hamster ovary cells [20] (clonogenic cell survival). The reason for the discrepancy is not clear. A number of mixed beam studies have been published, where the high and low LET doses were applied in sequence. The majority of studies were carried out with human or hamster epithelial cells, with clonogenic cell survival as the endpoint. 11 detected synergism [52–62] and 5 detected additivity [63–67]. It is obvious that factors other than the cell type and the analysed endpoint influence the outcome of a mixed beam study and it cannot be concluded that the effect is restricted to a particular cell type.

## Conclusions

The outcome of this study confirms earlier findings that alpha particles and X-rays interact in producing DNA damage above the level predicted by additivity. The result is interesting both from the mechanistic perspective of how cells cope with simultaneous clustered and dispersed DNA damage and from the perspective of radiation protection because they suggest that the health effects of mixed beam exposure may be higher than expected from additivity of the single mixed beam components. Additionally, the results are encouraging for future combination of high and low LET radiation in radiotherapy.

## Supporting information

S1 Table Fitting coefficients of the dose response curves and repair kinetics. (PDF)

S2 Table Fitting coefficients of the comet distributions. (PDF)
