# Supplementary material for: Simultaneous induction of dispersed and clustered DNA lesions compromises DNA damage response in human peripheral blood lymphocytes
Source: PLoS One. 2018 Oct 31;13(10):e0204068. doi: 10.1371/journal.pone.0204068 (PMC6209146; doi:10.1371/journal.pone.0204068)
Supplement: S1 Table — (PDF) [file pone.0204068.s001.pdf]

S1 Table. Fitting coefficients of the dose response curves and repair kinetics.

Type of result	Coefficients	Alpha particles	X-rays	Mixed beams
Dose response	a	3.56	10.59	11.89
	b	1.52	0.78	0.68
Repair kinetics	a	6.0×10^{-5}	1.1×10^{-5}	2.0×10^{-7}
	b	1.0×10^{-5}	2.7×10^{-6}	1.0×10^{-7}
	c	2.12	2.49	3.37
