# Supplementary material for: Simultaneous induction of dispersed and clustered DNA lesions compromises DNA damage response in human peripheral blood lymphocytes
Source: PLoS One. 2018 Oct 31;13(10):e0204068. doi: 10.1371/journal.pone.0204068 (PMC6209146; doi:10.1371/journal.pone.0204068)
Supplement: S2 Table — (PDF) [file pone.0204068.s002.pdf]

S2 Table. Fitting coefficients of the comet distributions.

Time (min)	Alpha particles		X-rays		Mixed beams	
	a	*b*	*a*	*b*	*a*	*b*
0	1.73 ± 0.07	7.1 ± 0.2	1.99 ± 0.06	12.7 ± 0.3	2.5 ± 1.0	18.6 ± 0.4
15	1.55 ± 0.03	6.69 ± 0.09	1.64 ± 0.06	8.2 ± 0.3	2.42 ± 0.10	13.1 ± 0.3
30	1.46 ± 0.03	5.28 ± 0.09	1.57 ± 0.06	7.4 ± 0.2	2.12 ± 0.08	12.1 ± 0.3
60	1.46 ± 0.06	5.64 ± 0.18	1.64 ± 0.04	5.81 ± 0.12	2.07 ± 0.07	11.7 ± 0.3
120	1.52 ± 0.03	5.38 ± 0.07	1.65 ± 0.05	5.73 ± 0.12	2.15 ± 0.06	9.94 ± 0.18
180	1.25 ± 0.04	4.18 ± 0.12	1.21 ± 0.05	5.4 ± 0.2	1.88 ± 0.06	9.9 ± 0.2
